# *catena*-Poly[[[diaqua­(1,10-phenanthroline-κ^2^ *N*,*N*′)cobalt(II)]-μ-4-hy­droxy-3-sulfonato­benzoato-κ^2^ *O* ^3^:*O* ^1^] sesquihydrate]

**DOI:** 10.1107/S1600536812018752

**Published:** 2012-05-02

**Authors:** Xiang-Qian Fang, Shan Gao, Seik Weng Ng

**Affiliations:** a Key Laboratory of Functional Inorganic Material Chemistry, Ministry of Education, Heilongjiang University, Harbin 150080, People’s Republic of China; b Department of Chemistry, University of Malaya, 50603 Kuala Lumpur, Malaysia; c Chemistry Department, Faculty of Science, King Abdulaziz University, PO Box 80203 Jeddah, Saudi Arabia

## Abstract

The 1,10-phenanthroline-chelated Co^II^ atom in the polymeric title compound, {[Co(C_7_H_4_O_6_S)(C_12_H_8_N_2_)(H_2_O)_2_]·1.5H_2_O}_*n*_, is connected to the sulfonate O atom of one 4-hy­droxy-3-sulfonato­benzoate dianion and to the carboxyl­ate O atom of another dianion. It is also coordinated by two water mol­ecules in a *trans*-CoN_2_O_4_ octa­hedral environment. The dianion links adjacent metal atoms into a chain running along [110]. The chains are linked by O—H⋯O hydrogen bonds into a three-dimensional network.

## Related literature

For the isotypic Mn^II^ derivative, see: Fang *et al.* (2011 ▶).
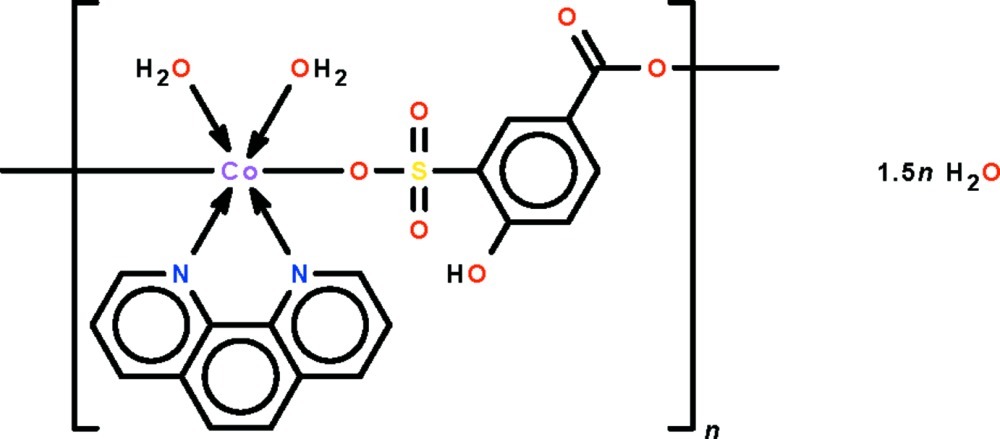


## Experimental

### 

#### Crystal data

[Co(C_7_H_4_O_6_S)(C_12_H_8_N_2_)(H_2_O)_2_]·1.5H_2_O
*M* *_r_* = 518.35
Monoclinic, 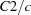

*a* = 8.3369 (4) Å
*b* = 17.3630 (6) Å
*c* = 28.6382 (10) Å
β = 93.189 (1)°
*V* = 4139.1 (3) Å^3^
*Z* = 8
Mo *K*α radiation
μ = 0.99 mm^−1^
*T* = 293 K
0.21 × 0.18 × 0.14 mm

#### Data collection

Rigaku R-AXIS RAPID IP diffractometer
Absorption correction: multi-scan (*ABSCOR*; Higashi, 1995 ▶) *T*_min_ = 0.819, *T*_max_ = 0.874
20042 measured reflections
4727 independent reflections
4053 reflections with *I* > 2σ(*I*)
*R*_int_ = 0.036

#### Refinement

*R*[*F* ^2^ > 2σ(*F* ^2^)] = 0.035
*wR*(*F* ^2^) = 0.096
*S* = 1.04
4727 reflections
326 parameters
8 restraints
H atoms treated by a mixture of independent and constrained refinement
Δρ_max_ = 0.48 e Å^−3^
Δρ_min_ = −0.23 e Å^−3^

### 

Data collection: *RAPID-AUTO* (Rigaku, 1998 ▶); cell refinement: *RAPID-AUTO*; data reduction: *CrystalClear* (Rigaku/MSC, 2002 ▶); program(s) used to solve structure: *SHELXS97* (Sheldrick, 2008 ▶); program(s) used to refine structure: *SHELXL97* (Sheldrick, 2008 ▶); molecular graphics: *X-SEED* (Barbour, 2001 ▶); software used to prepare material for publication: *publCIF* (Westrip, 2010 ▶).

## Supplementary Material

Crystal structure: contains datablock(s) global, I. DOI: 10.1107/S1600536812018752/bt5892sup1.cif

Structure factors: contains datablock(s) I. DOI: 10.1107/S1600536812018752/bt5892Isup2.hkl

Additional supplementary materials:  crystallographic information; 3D view; checkCIF report

## Figures and Tables

**Table 1:** Hydrogen-bond geometry (Å, °)

*D*—H⋯*A*	*D*—H	H⋯*A*	*D*⋯*A*	*D*—H⋯*A*
O4—H4⋯O4*W*	0.83 (1)	1.79 (1)	2.617 (2)	171 (3)
O1w—H11⋯O6^i^	0.85 (1)	1.69 (1)	2.526 (2)	169 (3)
O1w—H12⋯O3*W*	0.84 (1)	1.99 (1)	2.790 (1)	161 (2)
O2w—H21⋯O2^ii^	0.83 (1)	1.93 (1)	2.752 (2)	168 (3)
O2w—H22⋯O1*W*^iii^	0.83 (1)	1.93 (1)	2.756 (2)	171 (3)
O3w—H31⋯O2	0.85 (1)	1.92 (1)	2.754 (2)	167 (3)
O4w—H41⋯O4^iv^	0.83 (1)	2.29 (2)	2.952 (2)	137 (3)
O4w—H42⋯O5^v^	0.84 (1)	1.96 (1)	2.795 (2)	176 (3)

Symmetry codes: (i) 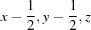; (ii) 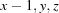; (iii) 
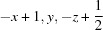
; (iv) 
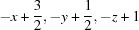
; (v) 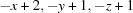.

**Fig. 1.**
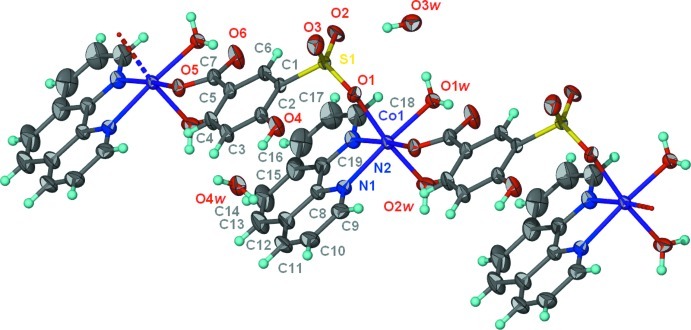
Anisotropic displacement ellipsoid plot (Barbour, 2001) of a portion of the structure of polymeric [Co(H2O)2(C12H8N2)(C7H4O6S)]n.1.5nH2O at the 50% probability level; hydrogen atoms are drawn as spheres of arbitrary radius.

## supplementary crystallographic information

### Comment

The title cobalt(II) compound (Scheme I, Fig. 1) is isostructural with the manganese(II) derivative, which we reported recently (Fang *et al.*, 2011). The 1,10-phenanthroline chelated Co^II^ atom is connected to the sulfonate O atom of one (C_7_H_4_O_6_S) dianion and to the carboxylate O atom of another dianion. It is also coordinated by two water molecules in an octahedral environment. The dianion links adjacent metal atoms into a chain along [1 1 0]. The chains are linked by O–H···O hydrogen bonds into a three-dimensional network (Table 1).

### Experimental

A methanol solution (5 ml) of 1,10-phenanthroline (1 mmol) was added to an aqueous solution (10 ml) of cobalt(II) dichloride (1 mmol), 2-hydroxy-5-carboxybenzenesulfonic acid (2 mmol) and lithium hydroxide (4 mmol). Pink crystals were isolated from the solution after several days.

### Refinement

All H atoms were located in a difference map. Carbon-bound H-atoms were placed in calculated positions (C–H 0.93 Å) and were included in the refinement in the riding model approximation, with *U*(H) set to 1.2*U*(C). H-atoms bonded to O were isotropically refined with a distance restraint of O–H 0.84±0.01 Å.

### Crystal data

**Table:**

[Co(C_7_H_4_O_6_S)(C_12_H_8_N_2_)(H_2_O)_2_]·1.5H_2_O	*F*(000) = 2128
*M**_r_* = 518.35	*D*_x_ = 1.664 Mg m^−^^3^
Monoclinic, *C*2/*c*	Mo *K*α radiation, λ = 0.71073 Å
Hall symbol: -C 2yc	Cell parameters from 16110 reflections
*a* = 8.3369 (4) Å	θ = 3.0–27.5°
*b* = 17.3630 (6) Å	µ = 0.99 mm^−^^1^
*c* = 28.6382 (10) Å	*T* = 293 K
β = 93.189 (1)°	Prism, pink
*V* = 4139.1 (3) Å^3^	0.21 × 0.18 × 0.14 mm
*Z* = 8	

### Data collection

**Table:**

Rigaku R-AXIS RAPID IP diffractometer	4727 independent reflections
Radiation source: fine-focus sealed tube	4053 reflections with *I* > 2σ(*I*)
Graphite monochromator	*R*_int_ = 0.036
ω scan	θ_max_ = 27.5°, θ_min_ = 3.0°
Absorption correction: multi-scan (*ABSCOR*; Higashi, 1995)	*h* = −10→10
*T*_min_ = 0.819, *T*_max_ = 0.874	*k* = −22→22
20042 measured reflections	*l* = −36→37

### Refinement

**Table:**

Refinement on *F*^2^	Primary atom site location: structure-invariant direct methods
Least-squares matrix: full	Secondary atom site location: difference Fourier map
*R*[*F*^2^ > 2σ(*F*^2^)] = 0.035	Hydrogen site location: inferred from neighbouring sites
*wR*(*F*^2^) = 0.096	H atoms treated by a mixture of independent and constrained refinement
*S* = 1.04	*w* = 1/[σ^2^(*F*_o_^2^) + (0.0593*P*)^2^ + 2.0124*P*] where *P* = (*F*_o_^2^ + 2*F*_c_^2^)/3
4727 reflections	(Δ/σ)_max_ = 0.001
326 parameters	Δρ_max_ = 0.48 e Å^−^^3^
8 restraints	Δρ_min_ = −0.23 e Å^−^^3^

### Fractional atomic coordinates and isotropic or equivalent isotropic displacement parameters (Å^2^)

**Table:**

	*x*	*y*	*z*	*U*_iso_*/*U*_eq_	
Co1	0.61702 (3)	0.233252 (12)	0.341796 (8)	0.02663 (9)	
S1	1.01602 (5)	0.26449 (2)	0.376637 (16)	0.02890 (11)	
O1	0.85592 (16)	0.23081 (7)	0.37033 (5)	0.0356 (3)	
O2	1.09098 (19)	0.27300 (8)	0.33199 (6)	0.0465 (4)	
O3	1.1137 (2)	0.22279 (8)	0.41122 (6)	0.0512 (4)	
O4	0.8735 (2)	0.31178 (7)	0.46465 (5)	0.0430 (3)	
O5	1.08477 (16)	0.62905 (7)	0.37595 (4)	0.0335 (3)	
O6	1.1048 (2)	0.54895 (8)	0.31558 (5)	0.0536 (4)	
O1W	0.69134 (16)	0.17451 (7)	0.28018 (4)	0.0316 (3)	
O2W	0.39077 (18)	0.22382 (11)	0.30945 (5)	0.0524 (4)	
O3W	1.0000	0.19799 (15)	0.2500	0.0502 (5)	
O4W	0.7312 (2)	0.35058 (9)	0.54035 (5)	0.0429 (3)	
N1	0.52131 (18)	0.30093 (9)	0.39551 (5)	0.0340 (3)	
N2	0.6524 (2)	0.34674 (9)	0.31625 (6)	0.0376 (3)	
C1	0.9907 (2)	0.35987 (9)	0.39700 (6)	0.0282 (3)	
C2	0.9196 (2)	0.37256 (10)	0.43952 (6)	0.0308 (4)	
C3	0.8980 (3)	0.44848 (11)	0.45451 (6)	0.0379 (4)	
H3	0.8491	0.4578	0.4824	0.045*	
C4	0.9488 (3)	0.50940 (10)	0.42819 (6)	0.0379 (4)	
H4A	0.9343	0.5595	0.4386	0.045*	
C5	1.0217 (2)	0.49711 (10)	0.38621 (6)	0.0314 (4)	
C6	1.0407 (2)	0.42151 (10)	0.37083 (6)	0.0308 (4)	
H6	1.0875	0.4125	0.3426	0.037*	
C7	1.0749 (2)	0.56273 (10)	0.35690 (6)	0.0337 (4)	
C8	0.5290 (2)	0.37789 (11)	0.38715 (7)	0.0386 (4)	
C9	0.4570 (3)	0.27742 (14)	0.43451 (7)	0.0437 (5)	
H9	0.4500	0.2248	0.4402	0.052*	
C10	0.3995 (3)	0.32857 (18)	0.46737 (8)	0.0594 (7)	
H10	0.3574	0.3102	0.4946	0.071*	
C11	0.4060 (3)	0.40557 (18)	0.45891 (9)	0.0651 (7)	
H11A	0.3665	0.4400	0.4803	0.078*	
C12	0.4721 (3)	0.43345 (14)	0.41810 (9)	0.0544 (6)	
C13	0.4873 (4)	0.51322 (16)	0.40639 (13)	0.0776 (10)	
H13	0.4504	0.5503	0.4266	0.093*	
C14	0.5536 (4)	0.53590 (14)	0.36690 (13)	0.0798 (9)	
H14	0.5605	0.5882	0.3604	0.096*	
C15	0.6139 (3)	0.48139 (13)	0.33467 (10)	0.0597 (7)	
C16	0.6870 (4)	0.50107 (15)	0.29369 (11)	0.0742 (9)	
H16	0.6988	0.5527	0.2859	0.089*	
C17	0.7411 (4)	0.44549 (15)	0.26510 (10)	0.0711 (8)	
H17	0.7908	0.4584	0.2379	0.085*	
C18	0.7202 (3)	0.36828 (13)	0.27749 (8)	0.0521 (6)	
H18	0.7556	0.3303	0.2576	0.063*	
C19	0.5997 (2)	0.40207 (11)	0.34500 (7)	0.0407 (4)	
H4	0.829 (3)	0.3290 (15)	0.4878 (6)	0.062 (8)*	
H11	0.665 (4)	0.1300 (9)	0.2888 (9)	0.072 (9)*	
H12	0.7900 (12)	0.1745 (13)	0.2762 (7)	0.037 (6)*	
H21	0.3019 (19)	0.2343 (14)	0.3198 (9)	0.054 (7)*	
H22	0.371 (3)	0.2041 (13)	0.2833 (5)	0.049 (7)*	
H31	1.022 (4)	0.2272 (13)	0.2731 (7)	0.064 (9)*	
H41	0.663 (3)	0.3175 (13)	0.5465 (10)	0.066 (9)*	
H42	0.789 (3)	0.3586 (17)	0.5647 (6)	0.070 (9)*	

### Atomic displacement parameters (Å^2^)

**Table:**

	*U*^11^	*U*^22^	*U*^33^	*U*^12^	*U*^13^	*U*^23^
Co1	0.03140 (14)	0.02203 (14)	0.02687 (14)	−0.00093 (8)	0.00532 (9)	−0.00072 (8)
S1	0.0284 (2)	0.0234 (2)	0.0354 (2)	−0.00200 (14)	0.00557 (17)	−0.00556 (16)
O1	0.0321 (6)	0.0297 (6)	0.0449 (7)	−0.0070 (5)	0.0020 (6)	−0.0050 (5)
O2	0.0537 (9)	0.0385 (8)	0.0502 (8)	−0.0041 (6)	0.0275 (7)	−0.0121 (6)
O3	0.0526 (9)	0.0364 (7)	0.0626 (10)	0.0058 (6)	−0.0151 (8)	−0.0030 (7)
O4	0.0706 (10)	0.0267 (6)	0.0337 (7)	−0.0100 (6)	0.0203 (7)	−0.0009 (6)
O5	0.0480 (7)	0.0227 (6)	0.0303 (6)	−0.0087 (5)	0.0069 (5)	−0.0014 (5)
O6	0.0967 (13)	0.0307 (7)	0.0358 (7)	−0.0174 (8)	0.0263 (8)	−0.0050 (6)
O1W	0.0364 (7)	0.0294 (6)	0.0298 (6)	−0.0029 (5)	0.0083 (5)	0.0005 (5)
O2W	0.0314 (7)	0.0876 (13)	0.0381 (8)	0.0092 (7)	−0.0003 (6)	−0.0220 (8)
O3W	0.0420 (12)	0.0638 (15)	0.0454 (12)	0.000	0.0063 (9)	0.000
O4W	0.0595 (9)	0.0395 (8)	0.0305 (7)	−0.0083 (7)	0.0088 (6)	0.0000 (6)
N1	0.0318 (8)	0.0364 (8)	0.0338 (7)	0.0031 (6)	0.0014 (6)	−0.0048 (7)
N2	0.0484 (9)	0.0272 (7)	0.0373 (8)	0.0003 (7)	0.0036 (7)	0.0030 (6)
C1	0.0325 (8)	0.0232 (7)	0.0290 (8)	−0.0046 (6)	0.0038 (6)	−0.0054 (6)
C2	0.0404 (9)	0.0252 (8)	0.0273 (8)	−0.0067 (7)	0.0061 (7)	−0.0014 (7)
C3	0.0566 (12)	0.0295 (9)	0.0291 (8)	−0.0072 (8)	0.0153 (8)	−0.0053 (7)
C4	0.0556 (12)	0.0248 (8)	0.0342 (9)	−0.0069 (8)	0.0113 (8)	−0.0068 (7)
C5	0.0411 (10)	0.0243 (8)	0.0292 (8)	−0.0085 (7)	0.0063 (7)	−0.0012 (7)
C6	0.0375 (9)	0.0270 (8)	0.0286 (8)	−0.0066 (7)	0.0086 (7)	−0.0039 (7)
C7	0.0417 (10)	0.0270 (8)	0.0331 (9)	−0.0079 (7)	0.0077 (7)	−0.0020 (7)
C8	0.0350 (9)	0.0353 (9)	0.0448 (10)	0.0081 (8)	−0.0038 (8)	−0.0124 (8)
C9	0.0369 (10)	0.0572 (12)	0.0374 (10)	0.0007 (9)	0.0064 (8)	−0.0043 (9)
C10	0.0449 (12)	0.090 (2)	0.0444 (12)	0.0010 (12)	0.0119 (9)	−0.0207 (12)
C11	0.0502 (13)	0.0847 (19)	0.0608 (14)	0.0121 (13)	0.0062 (11)	−0.0426 (14)
C12	0.0466 (12)	0.0519 (13)	0.0640 (14)	0.0140 (10)	−0.0033 (10)	−0.0260 (11)
C13	0.080 (2)	0.0485 (14)	0.103 (2)	0.0220 (14)	−0.0041 (18)	−0.0385 (16)
C14	0.107 (2)	0.0255 (11)	0.105 (2)	0.0165 (13)	−0.009 (2)	−0.0125 (14)
C15	0.0707 (17)	0.0281 (10)	0.0788 (17)	0.0043 (10)	−0.0089 (14)	0.0001 (11)
C16	0.105 (2)	0.0338 (12)	0.0823 (19)	−0.0094 (13)	−0.0047 (17)	0.0228 (13)
C17	0.106 (2)	0.0467 (14)	0.0613 (15)	−0.0139 (14)	0.0112 (15)	0.0195 (13)
C18	0.0737 (16)	0.0395 (11)	0.0439 (11)	−0.0062 (10)	0.0110 (11)	0.0070 (9)
C19	0.0442 (11)	0.0270 (9)	0.0500 (11)	0.0046 (7)	−0.0051 (9)	−0.0025 (8)

### Geometric parameters (Å, º)

**Table:**

Co1—O2W	2.0613 (15)	C2—C3	1.401 (2)
Co1—O5^i^	2.0813 (12)	C3—C4	1.379 (3)
Co1—O1	2.1107 (14)	C3—H3	0.9300
Co1—N1	2.1262 (15)	C4—C5	1.393 (2)
Co1—N2	2.1281 (15)	C4—H4A	0.9300
Co1—O1W	2.1586 (12)	C5—C6	1.396 (2)
S1—O3	1.4418 (16)	C5—C7	1.497 (2)
S1—O1	1.4592 (13)	C6—H6	0.9300
S1—O2	1.4616 (14)	C8—C12	1.410 (3)
S1—C1	1.7724 (16)	C8—C19	1.435 (3)
O4—C2	1.345 (2)	C9—C10	1.398 (3)
O4—H4	0.832 (10)	C9—H9	0.9300
O5—C7	1.275 (2)	C10—C11	1.360 (4)
O5—Co1^ii^	2.0813 (12)	C10—H10	0.9300
O6—C7	1.246 (2)	C11—C12	1.405 (4)
O1W—H11	0.845 (10)	C11—H11A	0.9300
O1W—H12	0.837 (10)	C12—C13	1.432 (4)
O2W—H21	0.833 (10)	C13—C14	1.345 (5)
O2W—H22	0.832 (10)	C13—H13	0.9300
O3W—H31	0.846 (10)	C14—C15	1.432 (4)
O4W—H41	0.832 (10)	C14—H14	0.9300
O4W—H42	0.837 (10)	C15—C16	1.395 (4)
N1—C9	1.330 (3)	C15—C19	1.415 (3)
N1—C8	1.360 (3)	C16—C17	1.359 (4)
N2—C18	1.327 (3)	C16—H16	0.9300
N2—C19	1.354 (3)	C17—C18	1.400 (3)
C1—C6	1.384 (2)	C17—H17	0.9300
C1—C2	1.401 (2)	C18—H18	0.9300
			
O2W—Co1—O5^i^	90.19 (7)	C3—C4—H4A	119.5
O2W—Co1—O1	173.02 (6)	C5—C4—H4A	119.5
O5^i^—Co1—O1	86.81 (5)	C4—C5—C6	118.59 (16)
O2W—Co1—N1	89.90 (6)	C4—C5—C7	121.63 (16)
O5^i^—Co1—N1	94.41 (5)	C6—C5—C7	119.76 (15)
O1—Co1—N1	96.61 (6)	C1—C6—C5	120.89 (15)
O2W—Co1—N2	93.43 (7)	C1—C6—H6	119.6
O5^i^—Co1—N2	172.07 (5)	C5—C6—H6	119.6
O1—Co1—N2	90.34 (6)	O6—C7—O5	124.64 (16)
N1—Co1—N2	78.56 (6)	O6—C7—C5	117.87 (16)
O2W—Co1—O1W	83.81 (6)	O5—C7—C5	117.48 (15)
O5^i^—Co1—O1W	91.37 (5)	N1—C8—C12	122.7 (2)
O1—Co1—O1W	89.96 (5)	N1—C8—C19	117.46 (16)
N1—Co1—O1W	171.47 (6)	C12—C8—C19	119.8 (2)
N2—Co1—O1W	96.03 (6)	N1—C9—C10	122.7 (2)
O3—S1—O1	111.20 (9)	N1—C9—H9	118.7
O3—S1—O2	113.52 (10)	C10—C9—H9	118.7
O1—S1—O2	111.40 (9)	C11—C10—C9	119.1 (2)
O3—S1—C1	108.44 (9)	C11—C10—H10	120.5
O1—S1—C1	106.87 (8)	C9—C10—H10	120.5
O2—S1—C1	104.95 (8)	C10—C11—C12	120.5 (2)
S1—O1—Co1	151.26 (9)	C10—C11—H11A	119.7
C2—O4—H4	107 (2)	C12—C11—H11A	119.7
C7—O5—Co1^ii^	126.22 (11)	C11—C12—C8	116.6 (2)
Co1—O1W—H11	96 (2)	C11—C12—C13	124.9 (2)
Co1—O1W—H12	116.1 (15)	C8—C12—C13	118.5 (3)
H11—O1W—H12	109 (3)	C14—C13—C12	121.7 (2)
Co1—O2W—H21	129.3 (19)	C14—C13—H13	119.1
Co1—O2W—H22	124.7 (18)	C12—C13—H13	119.1
H21—O2W—H22	106 (3)	C13—C14—C15	121.6 (2)
H41—O4W—H42	108 (3)	C13—C14—H14	119.2
C9—N1—C8	118.34 (17)	C15—C14—H14	119.2
C9—N1—Co1	128.54 (14)	C16—C15—C19	117.4 (2)
C8—N1—Co1	113.11 (12)	C16—C15—C14	124.4 (3)
C18—N2—C19	118.44 (18)	C19—C15—C14	118.2 (3)
C18—N2—Co1	128.46 (14)	C17—C16—C15	120.6 (2)
C19—N2—Co1	113.09 (13)	C17—C16—H16	119.7
C6—C1—C2	120.24 (15)	C15—C16—H16	119.7
C6—C1—S1	119.96 (13)	C16—C17—C18	118.5 (3)
C2—C1—S1	119.80 (13)	C16—C17—H17	120.7
O4—C2—C1	119.26 (15)	C18—C17—H17	120.7
O4—C2—C3	121.90 (15)	N2—C18—C17	123.1 (2)
C1—C2—C3	118.84 (15)	N2—C18—H18	118.4
C4—C3—C2	120.35 (16)	C17—C18—H18	118.4
C4—C3—H3	119.8	N2—C19—C15	122.0 (2)
C2—C3—H3	119.8	N2—C19—C8	117.78 (17)
C3—C4—C5	121.08 (16)	C15—C19—C8	120.2 (2)
			
O3—S1—O1—Co1	171.77 (16)	Co1^ii^—O5—C7—O6	10.2 (3)
O2—S1—O1—Co1	−60.5 (2)	Co1^ii^—O5—C7—C5	−168.98 (12)
C1—S1—O1—Co1	53.6 (2)	C4—C5—C7—O6	−164.0 (2)
O2W—Co1—O1—S1	115.4 (5)	C6—C5—C7—O6	14.1 (3)
O5^i^—Co1—O1—S1	−179.94 (18)	C4—C5—C7—O5	15.2 (3)
N1—Co1—O1—S1	−85.86 (18)	C6—C5—C7—O5	−166.62 (18)
N2—Co1—O1—S1	−7.34 (18)	C9—N1—C8—C12	0.2 (3)
O1W—Co1—O1—S1	88.68 (18)	Co1—N1—C8—C12	179.86 (16)
O2W—Co1—N1—C9	86.52 (17)	C9—N1—C8—C19	179.64 (17)
O5^i^—Co1—N1—C9	−3.66 (17)	Co1—N1—C8—C19	−0.6 (2)
O1—Co1—N1—C9	−90.95 (17)	C8—N1—C9—C10	−1.0 (3)
N2—Co1—N1—C9	−179.96 (18)	Co1—N1—C9—C10	179.36 (16)
O2W—Co1—N1—C8	−93.15 (14)	N1—C9—C10—C11	1.5 (4)
O5^i^—Co1—N1—C8	176.67 (13)	C9—C10—C11—C12	−1.1 (4)
O1—Co1—N1—C8	89.38 (13)	C10—C11—C12—C8	0.3 (4)
N2—Co1—N1—C8	0.37 (13)	C10—C11—C12—C13	−178.7 (3)
O2W—Co1—N2—C18	−92.3 (2)	N1—C8—C12—C11	0.2 (3)
O1—Co1—N2—C18	81.8 (2)	C19—C8—C12—C11	−179.3 (2)
N1—Co1—N2—C18	178.5 (2)	N1—C8—C12—C13	179.3 (2)
O1W—Co1—N2—C18	−8.2 (2)	C19—C8—C12—C13	−0.2 (3)
O2W—Co1—N2—C19	89.16 (14)	C11—C12—C13—C14	179.2 (3)
O1—Co1—N2—C19	−96.71 (14)	C8—C12—C13—C14	0.2 (4)
N1—Co1—N2—C19	−0.04 (14)	C12—C13—C14—C15	−0.6 (5)
O1W—Co1—N2—C19	173.29 (14)	C13—C14—C15—C16	−178.6 (3)
O3—S1—C1—C6	121.28 (16)	C13—C14—C15—C19	1.0 (5)
O1—S1—C1—C6	−118.75 (15)	C19—C15—C16—C17	0.3 (4)
O2—S1—C1—C6	−0.35 (18)	C14—C15—C16—C17	179.9 (3)
O3—S1—C1—C2	−58.89 (17)	C15—C16—C17—C18	0.7 (5)
O1—S1—C1—C2	61.08 (16)	C19—N2—C18—C17	0.5 (4)
O2—S1—C1—C2	179.48 (15)	Co1—N2—C18—C17	−178.0 (2)
C6—C1—C2—O4	−179.27 (17)	C16—C17—C18—N2	−1.1 (5)
S1—C1—C2—O4	0.9 (2)	C18—N2—C19—C15	0.5 (3)
C6—C1—C2—C3	1.0 (3)	Co1—N2—C19—C15	179.22 (17)
S1—C1—C2—C3	−178.80 (15)	C18—N2—C19—C8	−179.00 (19)
O4—C2—C3—C4	179.05 (19)	Co1—N2—C19—C8	−0.3 (2)
C1—C2—C3—C4	−1.3 (3)	C16—C15—C19—N2	−0.9 (4)
C2—C3—C4—C5	0.4 (3)	C14—C15—C19—N2	179.5 (2)
C3—C4—C5—C6	0.8 (3)	C16—C15—C19—C8	178.6 (2)
C3—C4—C5—C7	178.95 (19)	C14—C15—C19—C8	−1.0 (4)
C2—C1—C6—C5	0.1 (3)	N1—C8—C19—N2	0.6 (3)
S1—C1—C6—C5	179.94 (14)	C12—C8—C19—N2	−179.85 (19)
C4—C5—C6—C1	−1.0 (3)	N1—C8—C19—C15	−178.87 (19)
C7—C5—C6—C1	−179.22 (17)	C12—C8—C19—C15	0.6 (3)

Symmetry codes: (i) *x*−1/2, *y*−1/2, *z*; (ii) *x*+1/2, *y*+1/2, *z*.

### Hydrogen-bond geometry (Å, º)

**Table:**

*D*—H···*A*	*D*—H	H···*A*	*D*···*A*	*D*—H···*A*
O4—H4···O4*W*	0.83 (1)	1.79 (1)	2.617 (2)	171 (3)
O1w—H11···O6^i^	0.85 (1)	1.69 (1)	2.526 (2)	169 (3)
O1w—H12···O3*W*	0.84 (1)	1.99 (1)	2.790 (1)	161 (2)
O2w—H21···O2^iii^	0.83 (1)	1.93 (1)	2.752 (2)	168 (3)
O2w—H22···O1*W*^iv^	0.83 (1)	1.93 (1)	2.756 (2)	171 (3)
O3w—H31···O2	0.85 (1)	1.92 (1)	2.754 (2)	167 (3)
O4w—H41···O4^v^	0.83 (1)	2.29 (2)	2.952 (2)	137 (3)
O4w—H42···O5^vi^	0.84 (1)	1.96 (1)	2.795 (2)	176 (3)

Symmetry codes: (i) *x*−1/2, *y*−1/2, *z*; (iii) *x*−1, *y*, *z*; (iv) −*x*+1, *y*, −*z*+1/2; (v) −*x*+3/2, −*y*+1/2, −*z*+1; (vi) −*x*+2, −*y*+1, −*z*+1.
